# Diphenhydramine-Induced Torsade De Pointes With Pharmacological Cardioversion in a Patient With Methadone-Induced QT Prolongation

**DOI:** 10.7759/cureus.22534

**Published:** 2022-02-23

**Authors:** Jamie P Hoffman, Andrew A Serdiuk, Allan R Escher, Bruno Bordoni, Raymond Evans

**Affiliations:** 1 Anesthesiology, H. Lee Moffitt Cancer Center and Research Institute, Tampa, USA; 2 Anesthesiology/Pain Medicine, H. Lee Moffitt Cancer Center and Research Institute, Tampa, USA; 3 Physical Medicine and Rehabilitation, Don Carlo Gnocchi Foundation, Milan, ITA

**Keywords:** diphenhydramine, naranjo scale, pharmacologic cardioversion, magnesium, opioid use disorder (oud), chronic pain management, sevoflurane, qt interval prolongation, methadone, torsade de pointes

## Abstract

The anesthetic management of patients with chronic pain requires a thorough understanding of the physiologic changes resulting from long-term exposure to opioids, as well as a firm comprehension of the pharmacodynamic and pharmacokinetic properties of these medications. We present the case of a 60-year-old woman on methadone therapy presenting for cervical laminectomy and fusion. After intraoperative dysrhythmias, she underwent pharmacological cardioversion from torsade de pointes. This occurred intraoperatively after receiving 25 mg of intravenous diphenhydramine to attenuate erythema thought to be secondary to antibiotic administration. The use of a routine antihistamine may present a torsadogenic reaction in the setting of methadone maintenance treatment.

## Introduction

The perioperative management of patients with chronic pain is a formidable task confronting many anesthesiologists and pain physicians today. According to The Institute of Medicine, chronic pain affects about 100 million adults, accounts for $560-635 billion dollars annually in healthcare costs and lost productivity, and is the number one cause for missed work [1].

The QT interval is a measure of the time between the start of the Q wave and the end of the T wave in the heart’s electrical cycle. The QT interval represents electrical depolarization and repolarization of the ventricles. A prolonged QT interval (>500 ms) is a marker for potential ventricular tachyarrhythmias such as torsade de pointes (TdP) and is a risk factor for syncope and sudden death [2]. Methadone, the most widely used agent for opioid maintenance, may prolong the rate-corrected QT interval (QTc) and result in TdP [3]. According to The National Survey of Substance Abuse Treatment Services 2017 data, 382,867 patients enrolled in opioid treatment programs were receiving methadone for opioid dependence [4].

The H1-receptor antagonist diphenhydramine is a freely available, over-the-counter medication for sleep and the most frequently used antihistamine drug [5]. Most reports of diphenhydramine toxicity and associated QT interval prolongation involve high-dose administration up to 3 g in some case reports [5]. The extensive list of medications to avoid in patients with QT interval prolongation is contradictory in the anesthetic category, with volatile anesthetics representing drugs to avoid, when possible [6-8].

We report a case of a 60-year-old female, with methadone maintenance treatment, presenting for cervical laminectomy and fusion. After receiving 25 mg of diphenhydramine intravenously to treat erythema, thought to be secondary to antibiotic administration, she developed TdP. Subsequently, the patient underwent pharmacological cardioversion from TdP to normal sinus rhythm.

## Case presentation

A 60-year-old female with a history of spinal stenosis and myelopathy presented for a cervical decompression laminectomy with fusion. The patient had a medical history significant for smoking, multiple strokes without residual deficits, a carotid endarterectomy, alcohol use disorder, depression, and a potassium level of 3.0 mmol/L. For a short, outpatient surgery, a potassium cut-off level of 3.0 mmol/L was deemed clinically acceptable. She was taking methadone 0 mg every six hours for methadone maintenance of her chronic cervicalgia, along with escitalopram for depression. The patient took her last methadone 10 mg dose at bedtime before her nil per os (NPO) time. The preoperative electrocardiogram showed a heart rate of 103 beats per minute with occasional premature ventricular complexes and a QTc of 495 ms, which is considered prolonged beyond the borderline range between 440 ms and 460 ms in females. A serum magnesium level is not routinely obtained in the ambulatory surgery setting. The patient had been under general anesthesia with volatile anesthetics in the past without complications.

After receiving intravenous (IV) midazolam 2 mg, the patient was taken to the operating room and placed on standard American Society of Anesthesiologists (ASA) monitors. Induction of anesthesia commenced with IV lidocaine 60 mg, propofol 150 mg, and fentanyl 100 µg. After assuring successful mask ventilation, succinylcholine 60 mg was administered and endotracheal intubation was completed without difficulty.

**Figure 1:**
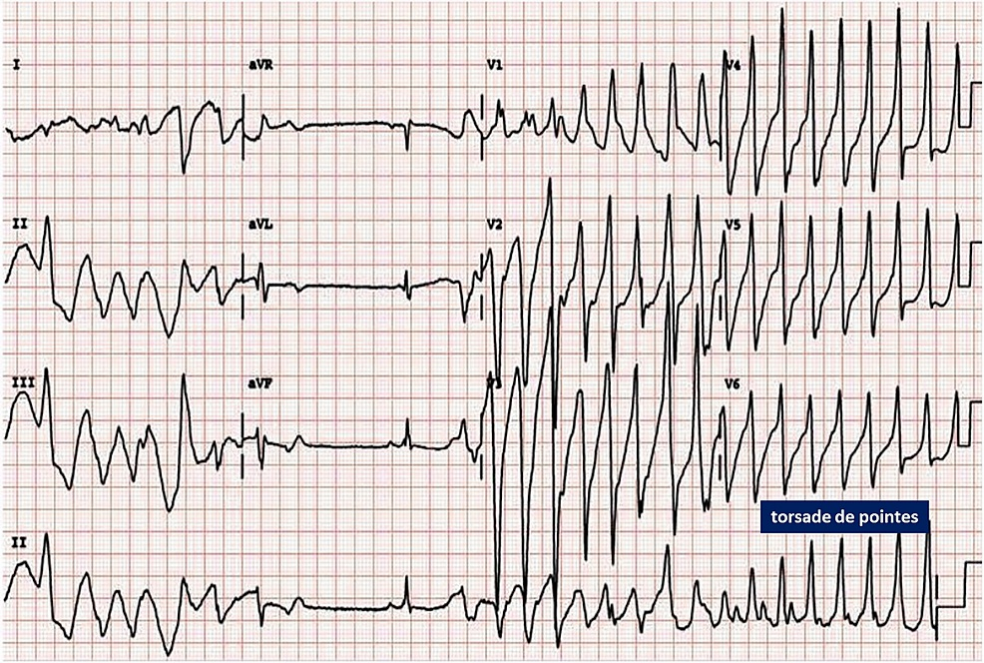
Electrocardiogram demonstrating TdP preceded by the classical short-long-short RR interval. TdP: torsades de pointes; RR: time elapsed between two successive R-waves of the QRS signal on the electrocardiogram

Preoperative prophylactic antibiotics, vancomycin and gentamicin, were then started and set to infuse over an hour. An additional 18-gauge IV cannula was placed as well as a radial arterial line. Maintenance of anesthesia was accomplished with intermittent 25 µg boluses of fentanyl and sevoflurane with 40% FiO_2_. The anesthetic proceeded uneventfully for the first two hours with infrequent doses of IV phenylephrine 100 µg for blood pressure support. Premature ventricular contractions continued sporadically. An episode of ventricular trigeminy prompted an electrocardiogram (ECG). While attaching the ECG leads, it was noted that the patient had developed an erythematous rash on the arm into which the vancomycin had been infused. To treat this reaction, IV diphenhydramine 25 mg was given. Her pulse rate was 56 beats per minute, her blood pressure was 108/54 mmHg, and her oxygen saturation was 98%. Shortly thereafter, following diphenhydramine administration, the patient’s heart rhythm changed into a wide-complex tachycardia with varying size, suspicious for TdP morphology (Figure 1).

A call for help was given, and the surgical team was notified. The FiO_2_ was increased to 100%. The volatile anesthetic was decreased, and auscultation was performed to ensure adequate ventilation. The Code Cart was brought into the operating room, and defibrillator pads were placed on the patient. At this time, the mean arterial blood pressure remained stable at 60-65 mmHg.

The arrhythmia was treated with magnesium 1 g, slow IV push, and IV lidocaine 100 mg, with the cardiac rhythm returning to a normal sinus rhythm. A magnesium infusion of 1 g IV over 15 minutes was given. The surgery was completed within an hour, and there were no more cardiac events or irregular rhythms during the perioperative course. The patient was taken to the recovery room in stable condition. Postoperative lab work showed a potassium level of 3.1 mmol/L, magnesium of 1.8 mg/dL, and calcium of 8.6 mg/dL. A cardiology consult was requested, and the patient was taken to the intensive care unit for further evaluation. A postoperative ECG showed a further increase in her QTc duration from the preoperative 495 ms to 607 ms. The methadone was discontinued for the duration of the patient’s hospital stay, and a follow-up ECG a week later showed that the QTc had normalized to 435 ms. The postoperative course was complicated by sepsis and respiratory failure requiring tracheostomy, and the patient was eventually discharged to a long-term care and rehabilitation facility.

## Discussion

The QT portion of the cardiac cycle involves a complex interplay of sodium and potassium flowing through various ion channels of the myocardium [9]. Due to the complexity of these channels, the QT interval is susceptible to perturbations in ion flow, which can prolong this portion of the cardiac cycle. The clinical results can range from simple prolongation such as long QT syndrome (LQTS) to malignant dysrhythmias, most notably TdP, and sudden death [9]. LQTS can be congenital, involving a mutation of an ion channel gene, or acquired as a result of several factors. Some of these factors include medications, synergistic drug interactions, electrolyte abnormalities, female sex, alcohol abstinence syndrome, and advanced age [10].

The list of drugs implicated in prolonging the QT interval is quite extensive, some of which the patient was taking before surgery and was given intraoperatively. Antihistamines, antidepressants, macrolide antibiotics, neuroleptic medications, inotropic/vasoconstrictive agents, anti-nausea medications, bronchodilators, and volatile anesthetics were given to the patient, which have all been implicated in the literature.

Preoperatively, the patient was on methadone maintenance treatment for cervicalgia, which has been found to interact with voltage-gated potassium channels of the myocardium. This can lead to prolonged action potentials and result in a prolonged QT interval. Viewig et al. found in their meta-analysis of TdP that as many as 80% of patients on long-term methadone therapy have a prolonged QT interval compared to equally matched controls [11]. They also stated that the risk of developing TdP increases with the presence of additional risk factors [11]. The patient’s methadone use alone was likely insufficient to elicit TdP but became clinically significant when combined with the synergistic effects of the other QT-prolonging medications received intraoperatively.

Diphenhydramine toxicity is dose-dependent. At higher concentrations, it inhibits the potassium channels responsible for repolarization, resulting in QT interval prolongation and abnormal ventricular repolarization. In the study by Zareba et al., in a retrospective analysis of 126 patients who attempted to commit suicide by overdosing on diphenhydramine, a significantly prolonged QTc developed after an average dose of 500 mg (453 ± 43 ms), compared to age and sex-matched controls (416 ± 35 ms) [12]. None of the patients reported in this study experienced TdP, though they did tend to have tachycardia, which may decrease risk [12].

Phenylephrine is commonly used to maintain blood pressure, either in boluses or by continuous infusion. Its mechanism as a peripheral alpha-agonist leads to vasoconstriction with an increase in systemic vascular resistance and afterload. This also typically leads to reflex bradycardia due to parasympathetic stimulation, which has been found to increase the QT interval and is a risk for developing TdP in some patients. Khositseth et al. found that patients with a subset of congenital LQTS receiving 2 µg/kg of phenylephrine intravenously demonstrated a significantly increased QTc [13].

Often overlooked causative agents responsible for QT prolongation in anesthetic practice are volatile anesthetic gases. Yildirim et al. found that all commonly used inhaled agents significantly prolonged the QTc interval above the baseline [7]. In particular, sevoflurane was found to have its maximal effect on the QT interval at approximately 10 minutes after reaching a steady level of one minimum alveolar concentration [7]. Alternatively, a prospective, randomized study of 60 patients, ASA I-II physical status, undergoing non-cardiac surgery revealed a significant increase in the QTc interval with the volatile anesthetic desflurane [8].

The pharmacodynamic effects of the various medications given, along with the patient’s multiple non-medication-related risk factors (advanced age, female sex, and hypokalemia), may have synergistically contributed to the prolonged QT interval and TdP. Based on the timing of events, diphenhydramine appears to have been the determinant etiology. Diphenhydramine 25 mg IV, a dose well below that correlated with the development of TdP, was sufficient, in this patient, to trigger an episode of TdP.

Magnesium sulfate IV, an effective antiarrhythmic, can be used successfully in the treatment of TdP in the hemodynamically stable patient; ideally, therapy should be guided in these patients based on a 12-lead ECG [14]. Of course, if such a patient becomes unstable, the anesthesiologist should proceed with unsynchronized cardioversion. Administration of magnesium IV or intraosseous may be accompanied by hypotension, respiratory depression, and central nervous system depression [14]. In a refractory case of TdP, point-of-care monitoring of ionized magnesium (iMg) was shown to terminate TdP with a target iMg set to 1.3 mmol/L [15].

## Conclusions

Chronic pain patients present challenges in the perioperative setting, most often with the planning for analgesic requirements. Of note, the widespread acceptance of methadone for the treatment of both chronic pain and opioid use disorder will result in ever-increasing numbers of such patients presenting for surgery. The use of familiar and common medications such as diphenhydramine may exert a sudden and pronounced effect on QT prolongation. A vigilant anesthesiologist must be cognizant of the torsadogenic effects of medications within the anesthetic armamentarium.
